# Inorganic Flame-Retardant Coatings Based on Magnesium Potassium Phosphate Hydrate

**DOI:** 10.3390/ma15155317

**Published:** 2022-08-02

**Authors:** Sin-Nan Chen, Ching Lin, Hao-Lun Hsu, Xin-Han Chen, Yu-Chang Huang, Tar-Hwa Hsieh, Ko-Shan Ho, Yu-Jun Lin

**Affiliations:** Department of Chemical and Materials Engineering, National Kaohsiung University of Science and Technology, Kaohsiung 807618, Taiwan; h722655@gmail.com (S.-N.C.); f110146145@nkust.edu.tw (C.L.); c108146220@nkust.edu.tw (H.-L.H.); c106146220@nkust.edu.tw (X.-H.C.); ych@nkust.edu.tw (Y.-C.H.); c108146243@nkust.edu.tw (Y.-J.L.)

**Keywords:** magnesium potassium phosphate hydrate-based flame-retardant coating, mullite whisker, flame-resistance limit, flame-resistance rating, ceramic shield, flame-retardancy

## Abstract

A magnesium potassium phosphate hydrate-based flame-retardant coating (MKPC) is formulated by dead-burnt magnesium oxide (magnesia) and potassium dihydrogen phosphate (KH_2_PO_4_), behaving as a matrix. Constituents of the MKPC include wollastonite, vermiculite, aluminum fluoride, aluminum trihydroxide, and calcium carbonate. Some of the ingredients inter-react to produce mullite whiskers at high temperatures, despite an acid-base hydration induced reaction between magnesia and KH_2_PO_4_. The MKPC’s thermal, corrosion-resistant, mechanical, and flame-resistant properties were analyzed using scanning electron microscopy, electrochemical corrosion testing, compression testing, thermogravimetric analysis, and freeze/thaw tests. The results show that with the molar ratio = 4 of magnesia to KH_2_PO_4_, MKPC demonstrates lower thermal conductivity (0.19 W/m K), along with better corrosion resistance, stronger compressive strength (10.5 MPa), and higher bonding strength (6.62 kgf/cm^2^) to the steel substrate. Furthermore, acceptable additives to the formulation could enhance its flame-retardancy and increase its mechanical strength as well. Mullite whisker formed from the interaction of wollastonite, aluminum trihydroxide, and aluminum fluoride acts as an outer ceramic shield that enhances mechanical strength and compactness. In addition, Mg-containing minerals with calcium carbonate treated at high temperatures, transform into magnesium calcium carbonate after releasing CO_2_. At the optimum composition of MKPC (magnesia/KH_2_PO_4_ molar ratio = 4; wollastonite:vermiculite = 20:10 wt.%; aluminum trihydroxide = 10 wt.%; and calcium carbonate = 5 wt.%), coated on a steel substrate, the flame-resistance limit results exhibit below 200 °C on the back surface of the steel substrate after one hour of flaming (ca. 1000 °C) on the other surface, and the flame-resistance rating results demonstrate only 420 °C on the back surface of the steel substrate after three hours of flaming (>1000 °C) on the other surface. Both requirements for the flame-resistance limit and three-hour flame-resistance rating are met with the optimum compositions, indicating that MKPC plays an effective role in establishing flame-retardancy.

## 1. Introduction

The topic of catastrophic fire has been around for decades, constantly causing massive loss of lives and resources. Therefore, passive flame-retardant coatings developed for steel-made construction materials play an important role in building construction. Generally, flame-retardant coatings are divided into organic [1] and inorganic [2] materials. The inorganic counterparts include intumescent [3] and non-intumescent coatings. In 2021, Chen et al. [4] reported a silicate-based intumescent flame-retardant coating, which showed remarkable results during firing. Even so, the practical requirements for water and weather resistance remain unmet. Therefore, silicate-based intumescent fire-retardant coatings are considered more suitable for interior decoration applications as they can meet most of the requirements.

Another possible alternative to inorganic matrices are hydraulic binding materials such as magnesium potassium phosphate cements, which are known for their high adhesion properties [5,6], good corrosion resistance to steel substrates [7,8], and freeze-thaw resistance. Furthermore, it has been reported that the rapid acid-base hydration reaction of MgO with KH_2_PO_4_ in aqueous solution is dominated by the water-cement ratio [9] and the MgO/KH_2_PO_4_ ratio [10,11], respectively.

Magnesium potassium phosphate cement has many excellent characteristics, such as rapid setting, high early strength, high fluidity, good volume stability, strong bonding strength to concrete substrates, and superior durability. Therefore, it has been applied in various fields such as the rehabilitation and repair of civil structures, the treatment of wastewater, and the stabilization of hazardous waste [12]. However, current studies on magnesium potassium phosphate cement mainly focus on the reaction and retardation mechanisms, the influencing factors on the properties of the magnesium potassium phosphate cement, and similar subjects. Some studies have shown that magnesium potassium phosphate cement has a high temperature resistance, but there are few reports on the use of magnesium potassium phosphate cement as a fire-retardant coating. Because most current fire-retardant coatings contain volatile organic components, they are expensive, toxic, and have low resistance to fire [5].

In the past, it has been reported in a small number of studies that magnesium potassium phosphate with flame-retardant properties can be used as a substrate for newly developed flame-retardant coatings [13,14]. However, these coatings also suffer from some disadvantages, such as poor mechanical strength and less flame-retardancy improvement in flame resistance, which demonstrates these coatings cannot easily be adapted to building material applications. Recently, Ding [5] suggested the incorporation of glass fiber or glass fiber powder as a mineral admixture into magnesium potassium phosphate cement for plywood fire-proof coating, which exhibited good spread fluidity, initial setting time, bonding strength, and fire-retardancy. CN 104230305 B [15] reported that a fire-proof coating based on magnesium potassium phosphate cement contains thermal insulation, inorganic material, and auxiliary agents, which suggests this coating can effectively replace traditional silicate cement and high-alumina cement for the inside wall of tunnels, to establish fire-retardancy. CN 104230305 B [16] described a fire-resistant coating based on magnesium potassium phosphate cement containing corundum sand, expanded perlite, and polypropylene fiber for steel structure coating. They pointed out this coating has a controllable setting time, hardens fast, cures at normal temperatures, is easy to preserve, is low-cost, has a higher bonding strength to the steel construction matrix, high mechanical strength, excellent fire-protecting performance, and good stability and endurance. Consequently, several mineral additives that can improve performance characteristics were selected in the formulations, including flame-resistance, toughness, mechanical strength, thermal shock resistance, weathering durability, etc. [17,18,19]. However, the thickness of magnesium potassium phosphate cement-based coatings layered in the substrate is still thicker (i.e., 15~20 mm), which may induce poor workability (slower construction and curing rate) and crack formation during the flame exposure. Despite these coatings meeting the requirements of the flame limit, which means that the magnesium potassium phosphate cement does not burn and that there is a time delay until the coated object begins to burn, it is very difficult to simulate the fire-retardancy of coatings under real flame conditions due to the lack of direct flame-resistance rating testing.

In consideration of these issues, our aim is to adopt ingredients that can be very effective in increasing the flame-retardancy of coatings for steel construction when exposed to flame. Additionally, proper thermal insulation additives (wollastonite and vermiculite) and functional additives (aluminum hydroxide, aluminum fluoride and calcium carbonate) selections are being implemented to enhance the anti-corrosion properties and overall mechanical strength. Therefore, as far as we understand, steel coated with the magnesium potassium phosphate hydrate-based flame-retardant coating (MKPC) can meet the requirements of steel construction for building material applications. In the present study, the MKPC’s fire-retardancy, corrosion behavior, and mechanical strength were analyzed by means of a flame-resistance limit and a flame-resistance rating test method, the Tafel method and the universal machine test method, respectively. For easy and fast construction and curing, a thinner MKPC coating was also adopted.

## 2. Materials and Methods

### 2.1. Preparation of Magnesium Potassium Phosphate Hydrate

Magnesium oxide (MgO 90%, SiO_2_ 5%, CaO 2.2%) and KH_2_PO_4_ (Taiwan Guosheng Plumbing Service Co., Ltd., Mailiao Township, Taiwan) were mixed in a molar ratio of 2–5 by a mechanical mixer. Water was then introduced during stirring at a weight ratio of 0.3 to the powder mixture. The resultant pastes prepared with various magnesia/KH_2_PO_4_ molar ratios were poured into molds and then cured at ambient temperatures.

### 2.2. Preparation of the MKPCs

The slurry described above contained mixtures of wollastonite (SiO_2_ 50.77%, CaO 45.26%, MgO 1.29%) and vermiculite ((MgFe,Al)_3_(Al,Si)_4_O_10_(OH)_2_∙4H_2_O 30% weight percentage) with ratios of 20:10, 10:20 and 10:10, respectively, acting as insulation additives. Functional additives included 4 wt.% aluminum fluoride (of AlF_3_∙31/2H_2_O, Emperor Chemical Co. Ltd., Taipei, Taiwan), 3~5 wt.% aluminum hydroxide (purity > 99%, particle size = 52 μm), and 3~10 wt.% calcium carbonate (ultra-pure reagent, precipitated). The MKPC pastes were prepared with a W/C ratio of 0.8 (W/C = 0.8), in which cement was based on the powder mixture. The MKPC was then brushed onto a steel substrate, creating a layer with a thickness of approximately 5 mm, and cured in the atmosphere.

### 2.3. Corrosion Characterization and Flame-Resistance Testing of the MKPCs

The chemical composition of the coating and the steel substrate, as well as the acronyms of the studied samples, are summarized in Table 1 and Table 2. SEM was used to characterize the surface morphology of the MKPC. A potentiostat was used to detect the electrochemical corrosion. The MKPC was first coated to one side of a 3 cm × 3 cm cleaned SN400YB steel substrate to a thickness of 5 mm, applied by brushing; the other side was then sealed with paraffin and fixed to a 1 cm × 1 cm soaking area. The MKPC specimens were immersed in a 3.5 wt.% NaCl (aq) soaking solution after 24 h of curing. The polarization curve of the MKPC specimens was analyzed using an Autolab potentiostat, with Ag/AgCl and platinum as the reference and counter electrode; the scan rate was 0.5 m Vs^−1^ over a potential range of between −300 and +1200 mV. The density of the instantaneous corrosion current density (J_cor_, expressed in mA cm^−2^) was calculated with Equation (1), and the corrosion rate, expressed as penetration rate (v_p_, expressed in mm/year), was calculated with Equation (2) [20,21].

J_cor_ = (b_a_ × b_c_)/(2.303 (b_a_ + b_c_) × R_p_) (1)

v_p_ = 3.27 × (A/Z) × (J_cor_/ρ) (2)

where b_a_ and b_c_ represent the slope of the linear portion of the anodic branch and the cathodic branch, respectively. In the diagram E = f (log J); R_p_ = (dE/dJ), E_cor_ is the polarisation resistance (expressed in Ω cm^2^), A/z represents the electrochemical equivalent of the corroding metal (in this case, iron was considered because it is the component with the largest amount in the alloy, A(Fe) = 55.85 g mol^−1^, and z = 2), and ρ is density (for Fe, ρ = 7.5 g cm^−3^). The crystal structure and phase change at high temperatures of the MKPC at different calcination levels were analyzed by a Bruker D8 Advance X-ray diffractometer (Karlsruhe, Germany). A thermogravimetric analyzer (Simultaneous DTA-TGA, SDT 2960, TA Instruments, New Castle, DE, USA), was used to evaluate the thermal stability of the MKPC. The thermal conductivity of the MKPC was obtained using a TechMax Technical Group Hot Disk Analyzer (TechMax Technical Co. Ltd., New Taipei, Taiwan). According to the Chinese specification (GB14907), the flammability limit of the MKPC (time required at ~250 °C) was found by using an alcohol blast burner at a temperature of ~1000 °C at a distance of ~6 cm from the MKPC. The thickness of the coating layered in the steel substrate was 5 mm, applied by brushing.

To find out the flame-retardancy rating, the prepared MKPC was adhered to a steel substrate and burned by a pilot flame (~1100 °C) at a distance of approximately 10 cm. A compressor machine with a dimension of 5 cm × 5 cm × 5 cm then tested the mechanical properties of the MKPCs.

## 3. Results and Discussion

### 3.1. Properties of the Magnesium Potassium Phosphate Hydrate Grown with Different Magnesia/KH_2_PO_4_ Molar Ratios

Magnesium oxide and KH_2_PO_4_ underwent an exothermic hydration reaction, and the chemical reaction schemes are shown below. The formation of MgKPO_4_ 6H_2_O (struvite-K) has a great influence on the solidification time, yield and mechanical strength of the resulting materials.

KH_2_PO_4_ → H_2_PO_4_^−^ + K^+^ (3)

H_2_PO_4_^−^ → H_2_PO_4_^−2^ + H^+^ (4)

MgO + H_2_O → Mg(OH)_2_ (5)

Mg(OH)_2_ → Mg^+2^_(aq)_ + 2OH^−^ (6)

Mg_(aq)_^2+^ + H_2_PO_4_^−^ + K^+^ + 6H_2_O → MgKPO_4_∙6H_2_O + 2H^+^ (7)

Mg_(aq)_^2+^ + HPO_4_^2−^ + K^+^ + 6H_2_O → MgKPO_4_∙6H_2_O + H^+^ (8)

**Figure 1:**
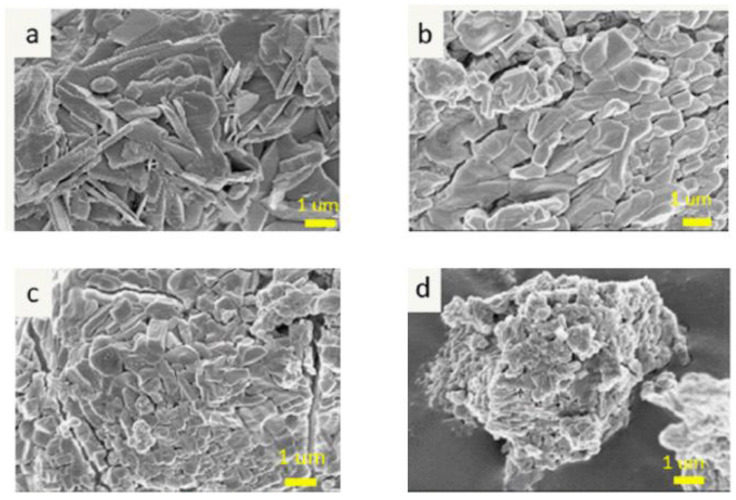
SEM images of MgKPO_4_·6H_2_O prepared with different magnesia/KH_2_PO_4_ molar ratios = (**a**) 2, (**b**) 3, (**c**) 4, and (**d**) 5.

Figure 1 shows the morphology of the hydrates formed in the hydration reaction with different MgO/KH_2_PO_4_ ratios. By mixing magnesium oxide and KH_2_PO_4_ with water, the acid-base reaction forms columnar Mg KH_2_PO_4_ 6H_2_O. Figure 1a shows that at the MgO/KH_2_PO_4_ molar ratio of two, the resulting hydrate has a sheet-like morphology with loose structure. When the ratio of MgO/KH_2_PO_4_ increases and reaches four, it leads to the formation of more MgKPO_4_∙6H_2_O (Figure 1b,c). When it exceeds four (Figure 1d), it can limit the hydration reaction with KH_2_PO_4_, resulting in spherical agglomeration.

**Figure 2:**
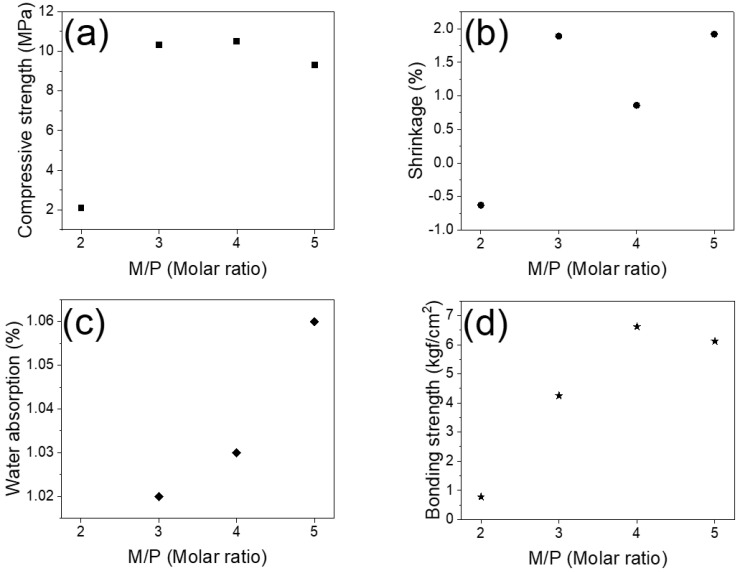
The properties of (**a**) compressive strength, (**b**) volume shrinkage, (**c**) water absorption, and (**d**) bonding strength of MgKPO_4_·6H_2_O at different magnesia/KH_2_PO_4_ molar ratios after a three-hour hydration reaction in the atmosphere.

The mechanical strength of the prepared hydrate is also related to the molar ratio of MgO/KH_2_PO_4_. Figure 2a shows that the compressive strength of the obtained MgKPO_4_·6H_2_O increases with the molar ratio, ranging from 2–4. At a molar ratio of four, the obtained maximum value of 10.5 MPa may be due to the additional hydration products produced during the hydration reaction. It indicates that the percentage of magnesium oxide interacting with KH_2_PO_4_ determines the final compressive strength. The release of water during the hydration process leads to the shrinkage of the surface and the generation of cracks inside the MgKPO_4_·6H_2_O. Figure 2b,c shows the effects of shrinkage and water absorption. Obviously, increasing the molar ratio can still cause shrinkage, even at a molar ratio of four. Likewise, as the molar ratio increases, the water absorption also increases. The adhesion between MgKPO_4_·6H_2_O and the steel matrix plays a crucial role in the tensile strength, as shown in Figure 2d. The most significant bond strength results from a better bonding strength of 6.62 kgf/cm^2,^ achieved at a molar ratio of four.

**Figure 3:**
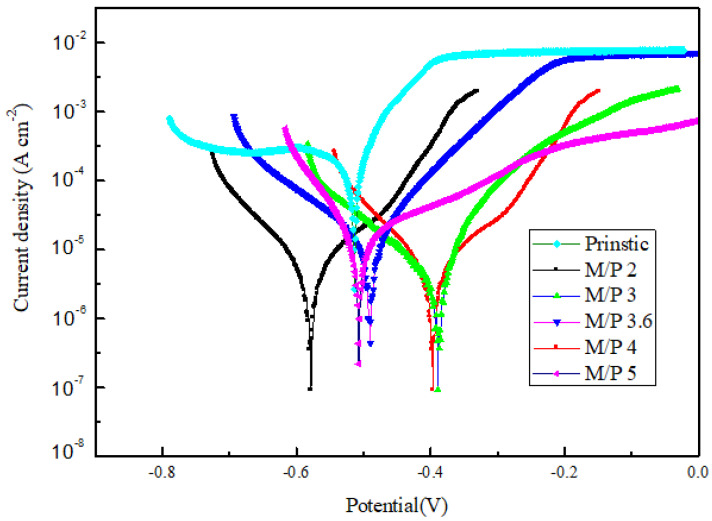
Potentiodynamic polarization for corrosion testing of MgKPO_4_·6H_2_O at different magnesia/KH_2_PO_4_ molar ratios.

The potentiodynamic polarization curves and various corrosion properties (i.e., corrosion potential, corrosion current density, b_a_, b_c_, polarization resistance and corrosion rate) of samples with different MgO/KH_2_PO_4_ molar ratios are shown in Figure 3 and Table 3, respectively. The results show that all samples have a negative value of the corrosion potential, which are relatively small changes, with values between −0.39 and −0.58 V. The corrosion current density, the polarization resistance and the corrosion rate present a large variation in values, between 2.79 and 28 μA cm^−2^, 0.82 and 19.1 (Ω cm^2^), and 0.033 and 0.34 (mm/year), respectively, which significantly reveals the corrosion behavior of the MKPCs varies with the MgO/KH_2_PO_4_ molar ratio. When the M/P molar ratio is three, the absolute value of corrosion potential, the corrosion current density and the corrosion rate obviously shifts to the lowest value, whereas the polarization resistance shifts towards the highest value, which demonstrates anti-corrosion tendency and the best corrosion resistance performance, due to the formation of a denser protective layer on the surface of steel substrate, contributed by phosphate and Fe^2+^ ions. It makes the surface of the substrate more resistant to corrosion. Various results including corrosion rate, corrosion potential, and corrosion current density, obtained at different MgO/KH_2_PO_4_ molar ratios, are listed in Table 3. Compared with the molar ratio four, molar ratio three is supposed to possess superior bonding strength. However, molar ratio four exhibited the best bond strength, as was seen in Figure 2d. Therefore, we speculate that the packaging condition within MgKPO_4_ 6H_2_O is influenced by the packaging density, which can create additional voids in the bulk specimen.

### 3.2. Influence of Thermal-Insulating Additives on the Flame Resistance of MKPCs

**Figure 4:**
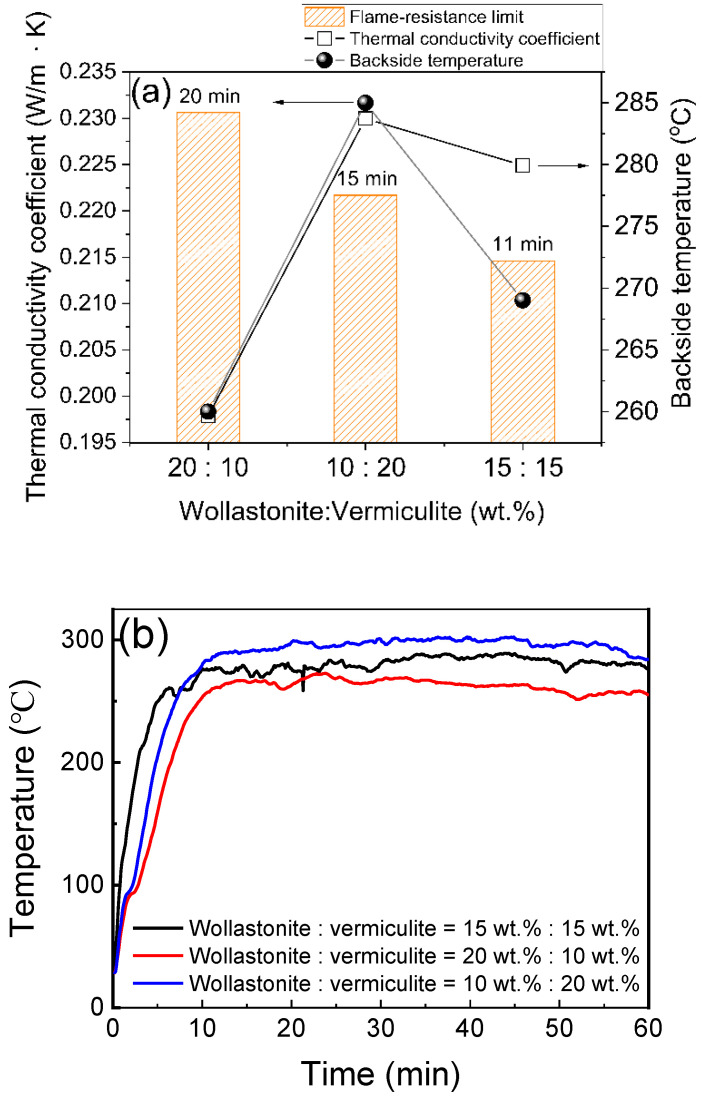
(**a**) Thermal conductivity, backside temperature for steel substrate, and flame-resistance limit; and (**b**) flame-resistance rating of the MKPCs with different weight ratios of wollastonite to vermiculite. (magnesia/KH_2_PO_4_ molar ratio = 4).

As is well known, magnesium potassium phosphate hydrate has moderate thermal-insulating properties. At high temperatures, the free water in the MKPC and the chemically-bonded water in the MgKPO_4_·6H_2_O dissipated a large amount of heat, which effectively retarded the spread of the fire and reduced the heat conduction of the coating. At M/P = 4 and W/C = 0.8, adding the additives, e.g., wollastonite, vermiculite, or aerogel with different contents, as in samples A1–A3, significantly enhanced the hydrate’s flame resistance. Figure 4a shows that, at a weight ratio of wollastonite to vermiculite of 20:10 (named sample A1), MKPC could reduce thermal propagation across the coating by a significant amount as it was under the invasion of flame. The lowered thermal conductivity of the MKPC is significantly attributed to the introduction of thermal-insulating additives in the coating. The results in Figure 4b also show that the flame-resistance rating of MKPCs can reach 250 °C within an hour after exposure to an alcohol blast burner flame. Using the optimum composition of wollastonite and vermiculite for the formulations, MKPCs reduce their thermal conductivity, and simultaneously enhance long-term flame-resistance.

**Figure 5:**
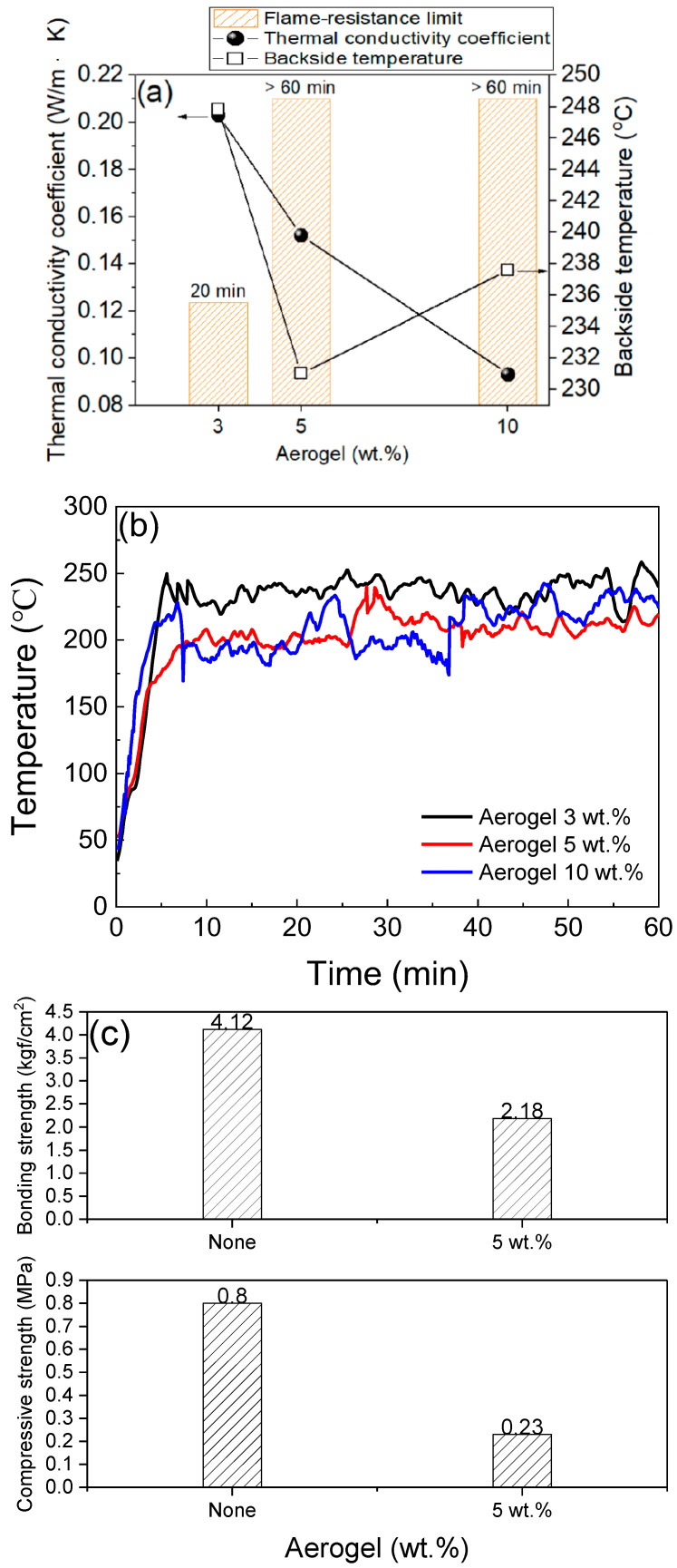
(**a**) Thermal conductivity, backside temperature for steel substrate, flame-resistance limit to different contents of aerogel and, (**b**) mechanical strength (**c**) bonding and compressive strength with 0 and 5 wt.% aerogel of the MKPCs. (magnesia/KH_2_PO_4_ molar ratio = 4; wollastonite:vermiculite = 20:10 wt.%).

Considering the high porosity, large surface area, and low thermal conductivity of aerogel, it could be used as a heat-capturing additive inside the MKPCs, as in samples B1–B3. As shown in Figure 5a, increasing the aerogel content leads to a gradual loss of the thermal conductivity of the MKPCs and an associated extension of the flame-resistance limit. The flame-retardancy shows that the backside temperature does not decrease linearly with decreasing thermal conductivity and increasing aerogel content. As shown in Figure 5b, the poor mechanical properties of MKPC samples A1 and B2, such as smaller adhesive strength and compressive strength, indicate that it lacks structural integrity to withstand suitable high-temperature combustion. As a result, aerogels are not considered ideal additives in MKPC formulations.

### 3.3. Mullite Whisker on the Flame Resistance and Microstructure of the MKPCs

**Figure 6:**
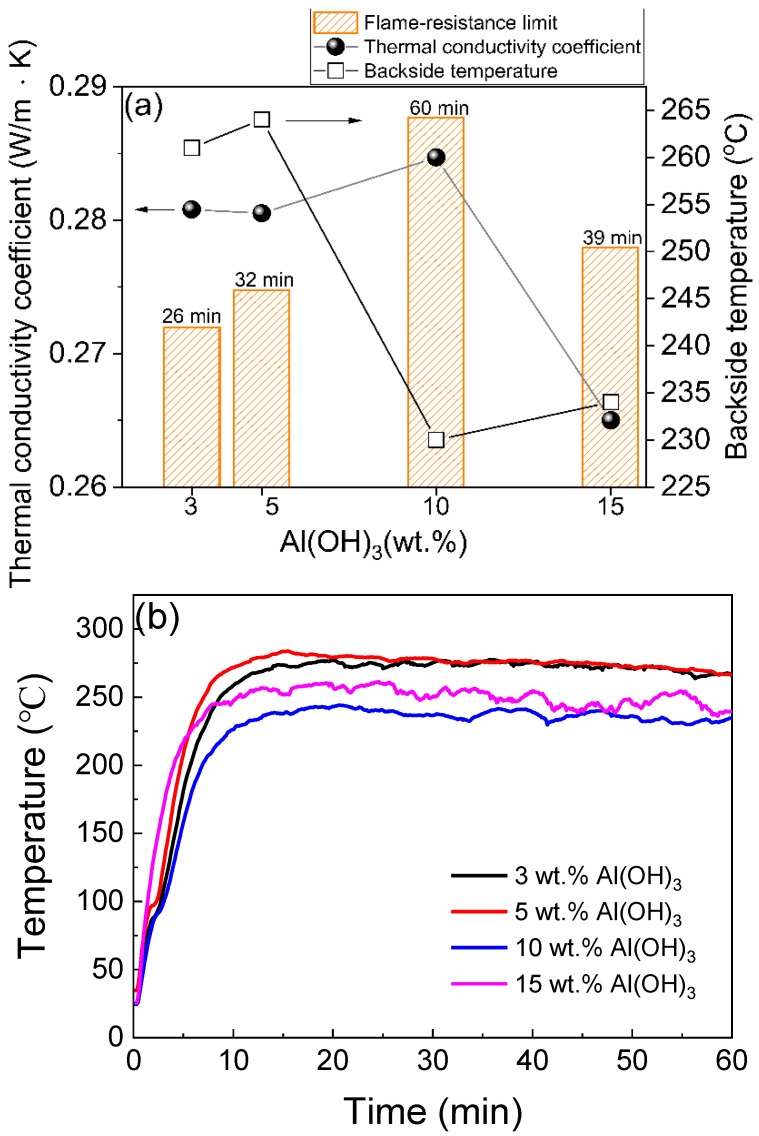
(**a**) Thermal conductivity, backside temperature for steel substrate, flame-resistance limit relating to different contents of aluminum trihydroxide, and (**b**) flame-resistance rating of the MKPCs. (magnesia/KH_2_PO_4_ molar ratio = 4; wollastonite:vermiculite = 20:10 wt.%).

Although the above-mentioned thermal insulation additives can enhance flame-retardancy, they still appear to affect the mechanical strength of MKPC. The carbon properties of mullite whiskers can act as heat shielding materials when exposed to flame, due to their high thermal stability, hardness and adhesive strength. The in situ growth of mullite whiskers requires active aluminum and silicon sources. Mullite whiskers are formed by combining wollastonite, aluminum hydroxide and aluminum fluoride at high temperatures. Figure 6a illustrates how MKPC samples C1–C4 extend their flame-retardancy limit by increasing the content of aluminum hydroxide. A backside temperature of 230 °C was obtained by adding aluminum hydroxide (10 wt.%) to the MKPC. In addition, the flame-retardancy ratings of different aluminum hydroxide contents of MKPC samples C1–C4 in Figure 6b are all below 275 °C, and it is considered that 10 wt.% aluminum hydroxide is the optimal level in the formulation. The thermal decomposition reaction of aluminum hydroxide is shown in Equation (7).

**Figure 7:**
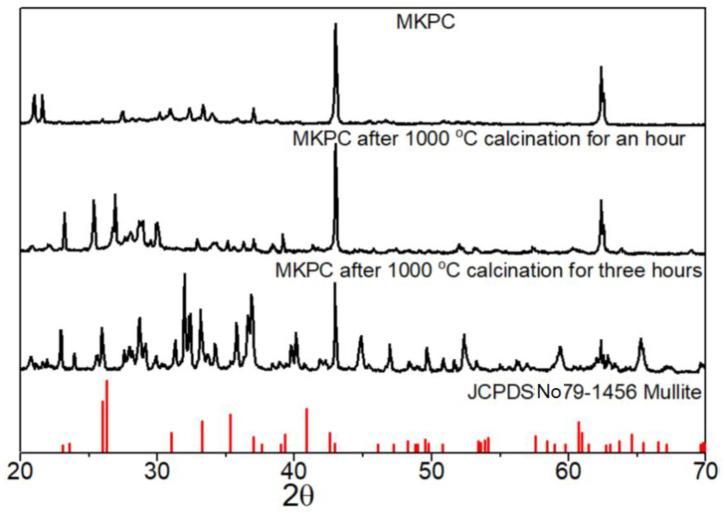
XRD diffractions of the MKPCs before and after the calcination at 1000 °C for one and three hours, respectively.

The XRD patterns in Figure 7, sample C3, demonstrate the formation of mullite whiskers after 1000 °C calcination for one and three hours, respectively, and indicate that mullite whiskers can generate at high temperatures. As shown in the figure, the diffraction peaks of magnesium oxide and MgKPO_4_·6H_2_O are located at 43°C and 22.5°C, respectively, and the characteristic peak of mullite appears at 28°C after calcination [19,22]. It means that aluminum hydroxide decomposes to alumina, which reacts with wollastonite to form mullite whiskers. Chemical reaction Equations (9)–(11) show how mullite is formed at ~900 °C.

2Al(OH)_3_ → Al_2_O_3_ + 3H_2_O (9)

Al(OH)_3_∙2H_2_O + 2SiO_2_ → Al_2_O_3_∙2SiO_2_ + 2H_2_O (10)

3Al_2_O_3_ + SiO_2_ → 3Al_2_O_3_∙2SiO_2_ (mullite) (11)

**Figure 8:**
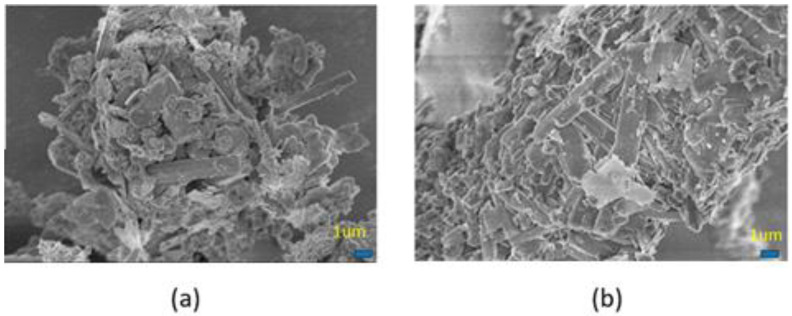
SEM images of the MKPCs (**a**) before, and (**b**) after calcination at 1000 °C for an hour. (magnesia/KH_2_PO_4_ molar ratio = 4; wollastonite:vermiculite = 20:10 wt.%; aluminum trihydroxide = 10 wt.%).

Compared with samples not exposed to an alcohol blast burner flame, Figure 8b exhibits more compact surface morphology than that illustrated in Figure 8a, sample C3, providing an indirect explanation for the formation of mullite whiskers at high temperatures.

### 3.4. Influence of Flame-Retardant Minerals of the MKPCs

**Figure 9:**
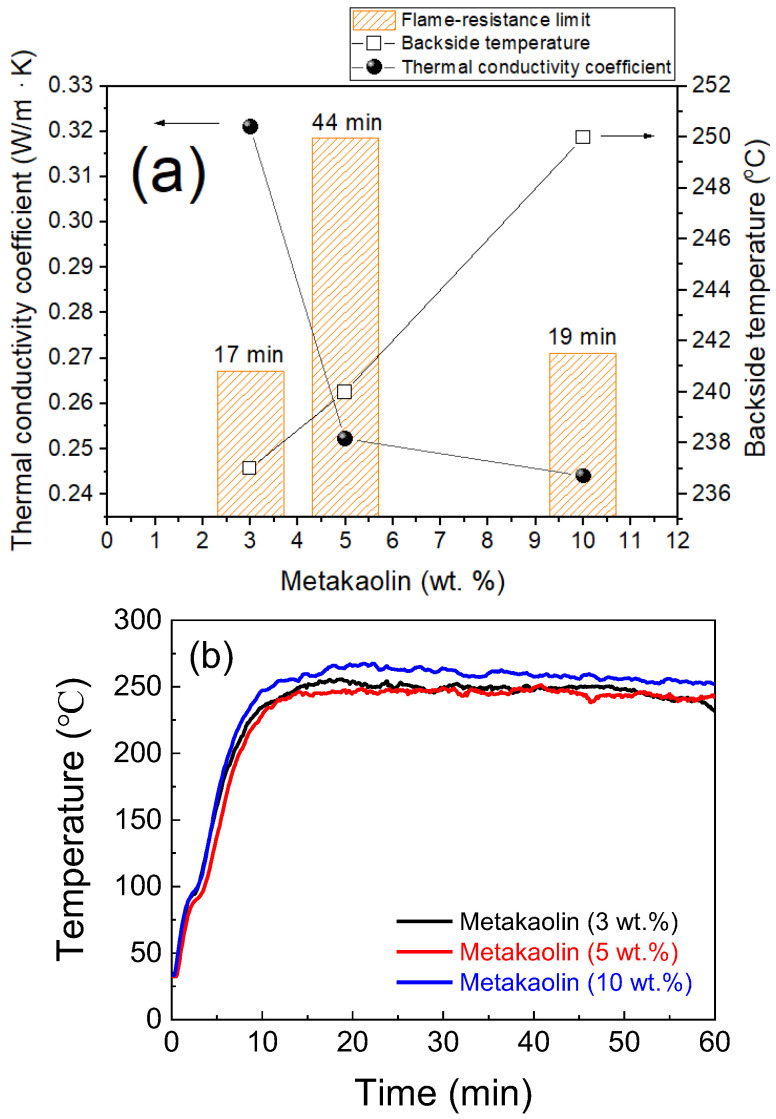
(**a**) Thermal conductivity, backside temperature for steel substrate, flame-resistance limit with respect to different contents of metakaolin, and (**b**) flame-resistance rating of the MKPCs. (magnesia/KH_2_PO_4_ molar ratio equals to 4; wollastonite:vermiculite = 20:10 wt.%; aluminum trihydroxide = 10 wt.%).

**Figure 10:**
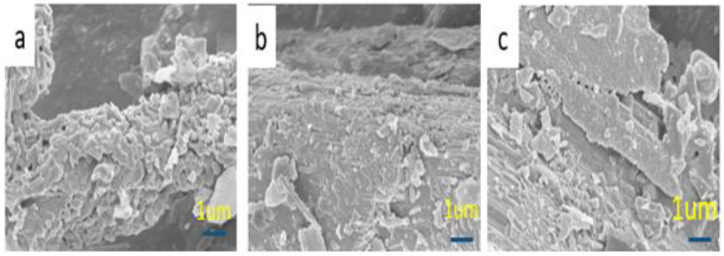
SEM surface images of the MKPCs with different contents of metakaolin: (**a**) 3, (**b**) 5, and (**c**) 10 wt.% after calcination at 1000 °C for an hour.

Certain minerals can effectively improve the fire resistance and mechanical properties of MKPC during flame exposure. The mineral metakaolin is used as a flame-retardant additive in MKPC formulations, considering its high flame-retardancy, flexibility and adhesion. Figure 9a shows that the thermal conductivity of MKPC samples D1–D3 decreases with the content of metakaolin. Theoretically, the thermal conductivity of MKPC is inversely proportional to the backside temperature. The corresponding fire-resistance limit first increases and then decreases. With the addition of 5% metakaolin, the backside temperature of MKPC reached around 240 °C, and its flame-retardancy limit and thermal conductivity were 44 min and 0.2522 w/m∙K, respectively. However, the flame-retardancy ratings in Figure 9b show that the increase in flame-retardancy of MKPC samples D1–D3 is not linear with the amount of metakaolin. From the morphology observed after heating at 1000 °C for one hour, Figure 10 shows that as MKPC samples D1–D3 had more metakaolin added, the denser their surface became. These results suggest that metakaolin is not suitable in formulating MKPCs as flame-retardants.

Alternatively, calcium carbonate can react with magnesium oxide to generate calcium magnesium carbonate, and its chemical reaction equation is as follows:

MgO + Ca(CO)_3_ → MgCa(CO_3_)_2_ (12)

**Figure 11:**
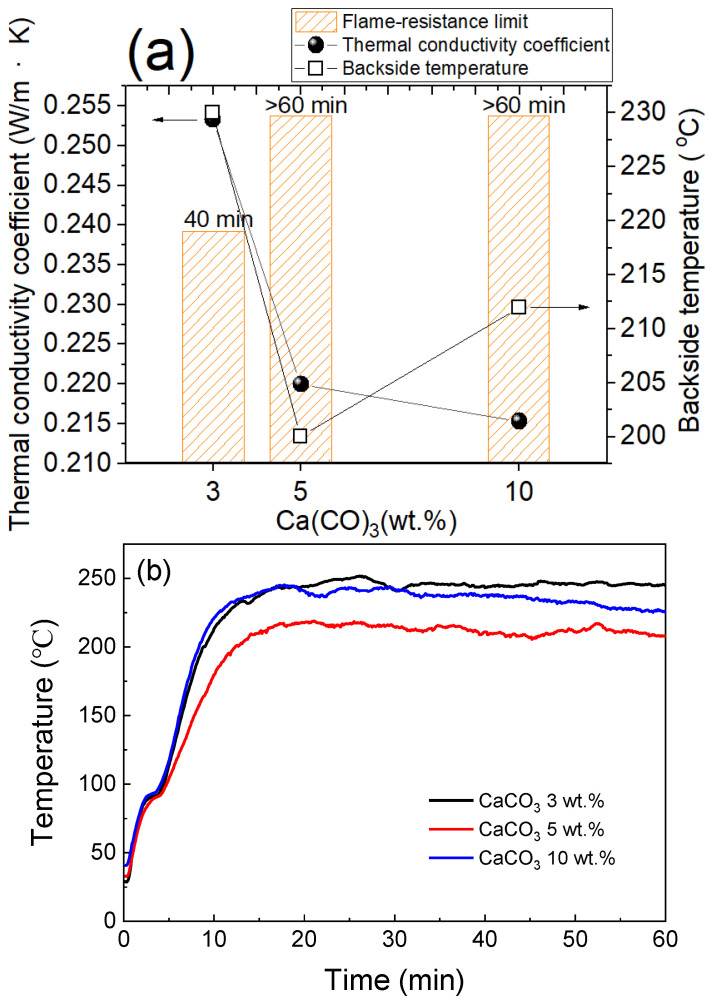
(**a**) Thermal conductivity, backside temperature, flame-resistance limit with respect to different contents of calcium carbonate, and (**b**) flame-resistance rating of the MKPCs (magnesia/KH_2_PO_4_ molar ratio = 4; wollastonite:vermiculite = 20:10 wt.%; aluminum trihydroxide = 10 wt.%).

**Figure 12:**
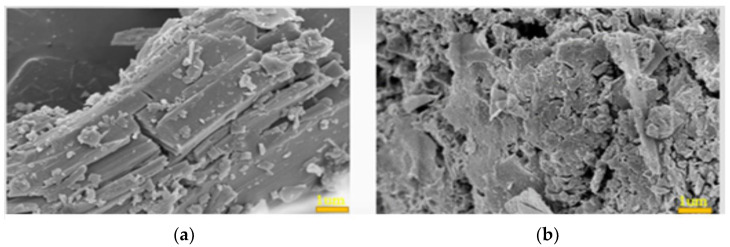
SEM images of flame-retardant coatings (**a**) before, and (**b**) after heating to 1000 °C for an hour. (magnesia/KH_2_PO_4_ molar ratios = 4; wollastonite:vermiculite = 20:10 wt.%; aluminum trihydroxide = 10 wt.%; calcium carbonate = 5 wt.%).

Calcium magnesium carbonate is relatively thermally stable and can decompose to carbon dioxide at high temperatures, thereby significantly reducing heat transfer from the flame to the substrate. Figure 11a shows that when the calcium carbonate content exceeds 5 wt.%, as in samples E1–E3, the flame-retardancy limit exceeds one hour. This is especially seen in the case of 5 wt.% calcium carbonate, where the thermal conductivity of MKPC is the lowest, at 0.2200 w/m∙K, and the backside temperature is approximately 200 °C. Figure 12 shows that MKPC samples E1–E3, containing calcium carbonate, became denser than neat one hour after heating at 1000 °C. However, excess calcium carbonate will form excess calcium oxide, which will further react with phosphoric acid to form tricalcium phosphate, thereby inhibiting the formation of MgKPO_4_·6H_2_O.

### 3.5. Physical Properties of the MKPCs with the Optimum Additives

**Figure 13:**
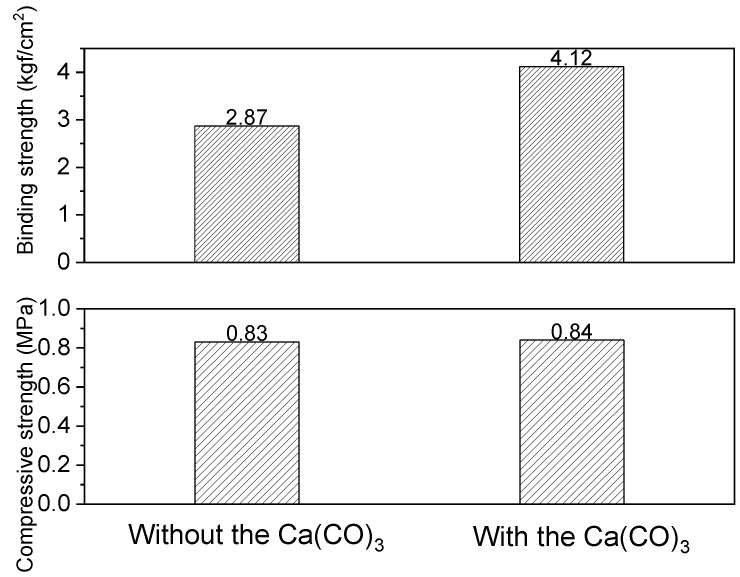
Mechanical properties of bonding and compressive strength of the optimum compositions of the MKPCs.

**Figure 14:**
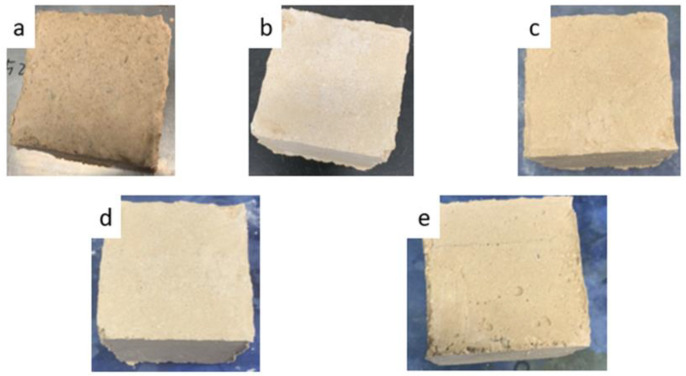
MKPCs with the optimum compositions (**a**) before and after (**b**) 1, (**c**) 5, (**d**) 10, and (**e**) 15 times freeze-thaw cycles.

**Figure 15:**
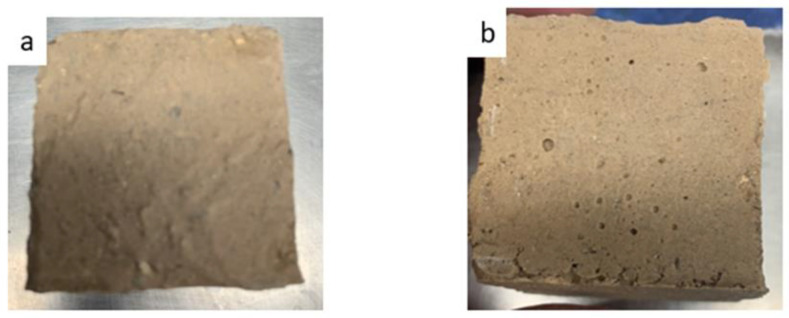
MKPCs with the optimum compositions (**a**) before, and (**b**) after water resistance testing for one day.

When developing MKPC, some basic properties such as mechanical properties, weather-resistance and flame-resistance need to be taken into consideration. The MKPC exhibited better mechanical properties when calcium carbonate was added (as per sample E2), as shown in Figure 13. Figure 14 shows visual images of MKPC sample E2 after different freeze-thaw cycles to investigate the degree of degradation under extreme conditions. None of the samples showed severe changes or deterioration such as visible cracks, peeling or blistering. The optimal composition of MKPC reduced deterioration, and sample E2 even remained intact when performing a water absorption test (Figure 15). The compact configuration of bulk samples allows minimal water vapor penetration into small voids, accumulating within the MKPC.

**Figure 16:**
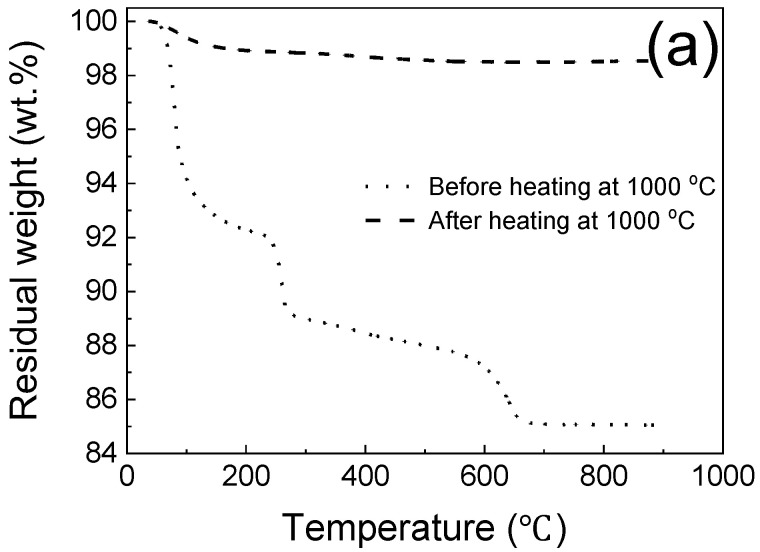

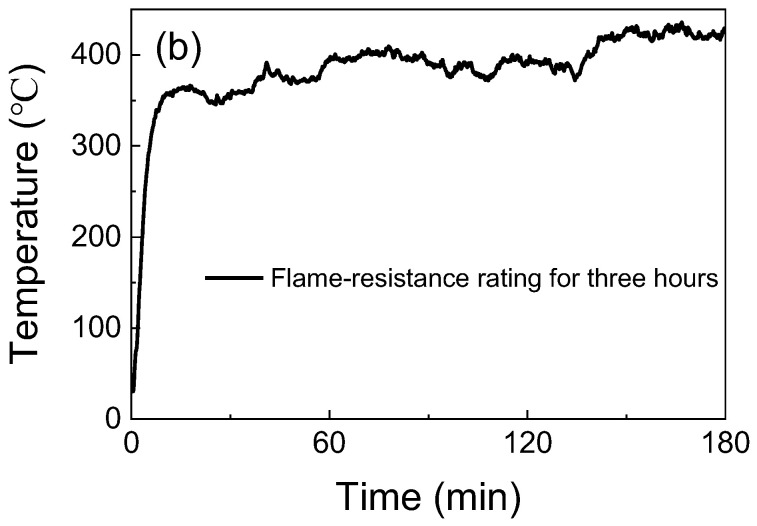
(**a**) Thermal analysis of the MKPCs before and after heating at 1000 °C, and (**b**) three hours of flame-resistance rating of the MKPC with the optimum compositions.

In Figure 16a, the thermal stability of MKPC sample E2 at elevated temperatures was studied by thermogravimetric analysis, which revealed several stages of weight loss during heating. Three corresponding weight losses occured at temperatures below 200 °C, 200~300 °C and 600~700 °C, respectively, with the loss of ions/water and crystallization of the main hydration products, the endothermic decomposition of aluminum trihydroxide, and CO_2_ release from calcium carbonate at high temperatures, respectively. The total residual weight loss of the MKPC after heating at 1000 °C was only 1.2 wt.%, while untreated MKPC was as high as 15 wt.%. This can be attributed to the formation of mullite whiskers in the MKPC, improving thermal stability, mechanical strength and flame-retardancy. As shown in Figure 16b, the three-hour FR rating of the best MKPC could maintain at 420 °C throughout. Therefore, the developed MKPCs can have remarkable flame-retardant properties.

## 4. Conclusions

The MKPC matrix is based on MgKPO_4_·6H_2_O. The composite material contains thermal insulation additives (wollastonite and vermiculite) and functional additives (aluminum hydroxide, aluminum fluoride and calcium carbonate). When the MgO/KH_2_PO_4_ molar ratio is four, the original compressive strength is 10.5 MPa and the bond strength is 6.62 kgf/cm^2^, respectively. Furthermore, adding the proper amount of wollastonite, vermiculite, aluminum trihydroxide and calcium carbonate to the formulation could enhance its flame-retardancy and increase mechanical strength as well. When exposed to flame, mullite whiskers enhance shielding strength and flame-retardancy by interacting with wollastonite, aluminum trihydroxide, and aluminum fluoride at 900 °C. At the optimum composition of MKPC (MgO/KH_2_PO_4_ molar ratio = 4; wollastonite:vermiculite = 20:10 wt.%; aluminum trihydroxide = 10 wt.%; and calcium carbonate = 5 wt.%), coated on a steel substrate, the flame-resistance limit is as low as ~200 °C and the three hours flame-retardant rating reaches approximately 420 °C. In the present study, the optimum MKPC coated steel substrate provided lower thermal conductivity, better corrosion resistance, stronger compressive strength, higher bonding strength, better resistance to freezing/thawing and water, as well as excellent flame-retardancy. Despite this, the application of actual steel structure fire protection, the steel type and composition, coating characteristics and thickness, the adhesion between the coating with primer and topcoat, as well as mechanical properties and weather resistance etc., must be considered precisely, and further research is required.

## Figures and Tables

**Table 1:** The composition of the MKPC samples at M/P = 4 and W/C = 0.8.

Sample	Wollastonite (wt.%)	Vermiculite (wt.%)	Aerogel (wt.%)	Aluminum Fluoride (wt.%)	Aluminum Hydroxide (wt.%)	Metakaolin (wt.%)	Calcium Carbonate (wt.%)
A1	20	10	-	-	-	-	-
A2	10	20	-	-	-	-	-
A3	15	15	-	-	-	-	-
B1	20	10	3	-	-	-	-
B2	20	10	5	-	-	-	-
B3	20	10	10	-	-	-	-
C1	20	10	-	4	3	-	-
C2	20	10	-	4	5	-	
C3	20	10	-	4	10	-	-
C4	20	10	-	4	15	-	-
D1	20	10	-	4	10	3	-
D2	20	10	-	4	10	5	-
D3	20	10	-	4	10	10	-
E1	20	10	-	4	10	-	3
E2	20	10	-	4	10	-	5
E3	20	10	-	4	10	-	10

**Table 2:** The composition of SN400YB, used as the steel substrate.

Composition	C	Si	Mn	P	S	B	Fe
Measured (wt.%)	0.2	0.35	0.6	0.03	0.015	0.0008	Balance

**Table 3:** Calculated parameters of MgKPO_4_·6H_2_O at different magnesia/KH_2_PO_4_ molar ratios.

Sample	E_corr_ (V vs. SCE)	J_corr_ (μA cm^−2^)	b_a_	b_c_	R_p_ ^a^ (Ω cm^2^)	v_p_ ^b^ (mm/year)
Pristine	−0.52	28	20.2	−32.8	0.82	0.340
MP = 2	−0.58	6	9.0	−22.6	1.08	0.073
M/P = 3	−0.39	2.79	18.0	−21.1	19.1	0.033
M/P = 3.6	−0.49	4.9	12.4	−20.5	2.7	0.059
M/P = 4	−0.39	3.29	13.1	−17.7	6.7	0.040
M/P = 5	−0.50	4.9	13.2	−22.8	2.8	0.059

^a^: data obtained from Equation (1). ^b^: data obtained from Equation (2).

## Data Availability

Not applicable.
